# Prostaglandin E Receptor Subtype EP3 Expression in Human Conjunctival Epithelium and Its Changes in Various Ocular Surface Disorders

**DOI:** 10.1371/journal.pone.0025209

**Published:** 2011-09-22

**Authors:** Mayumi Ueta, Chie Sotozono, Norihiko Yokoi, Tsutomu Inatomi, Shigeru Kinoshita

**Affiliations:** 1 Department of Ophthalmology, Kyoto Prefectural University of Medicine, Kyoto, Japan; 2 Research Center for Inflammation and Regenerative Medicine, Faculty of Life and Medical Sciences, Doshisha University, Kyoto, Japan; Alcon Research, Ltd., United States of America

## Abstract

**Background:**

In our earlier genome-wide association study on Stevens-Johnson Syndrome (SJS) and its severe variant, toxic epidermal necrolysis (TEN), we found that in Japanese patients with these severe ocular surface complications there was an association with prostaglandin E receptor 3 (EP3) gene (*PTGER3*) polymorphisms. We also reported that EP3 is dominantly expressed in the ocular surface-, especially the conjunctival epithelium, and suggested that EP3 in the conjunctival epithelium may down-regulate ocular surface inflammation. In the current study we investigated the expression of EP3 protein in the conjunctiva of patients with various ocular surface diseases such as SJS/TEN, chemical eye burns, Mooren’s ulcers, and ocular cicatricial pemphigoid (OCP).

**Methodology/Principal Findings:**

Conjunctival tissues were obtained from patients undergoing surgical reconstruction of the ocular surface due to SJS/TEN, chemical eye burns, and OCP, and from patients with Mooren's ulcers treated by resection of the inflammatory conjunctiva. The controls were nearly normal human conjunctival tissues acquired at surgery for conjunctivochalasis. We performed immunohistological analysis of the EP3 protein and evaluated the immunohistological staining of EP3 protein in the conjunctival epithelium of patients with ocular surface diseases. EP3 was expressed in the conjunctival epithelium of patients with chemical eye burns and Mooren’s ulcer and in normal human conjunctival epithelium. However, it was markedly down-regulated in the conjunctival epithelium of SJS/TEN and OCP patients.

**Conclusions:**

We posit an association between the down-regulation of EP3 in conjunctival epithelium and the pathogenesis and pathology of SJS/TEN and OCP, and suggest a common mechanism(s) in the pathology of these diseases. The examination of EP3 protein expression in conjunctival epithelium may aid in the differential diagnosis of various ocular surface diseases.

## Introduction

Prostanoids are comprised of prostaglandins (PGs) and thromboxanes (TXs). They are lipid mediators that form in response to various stimuli and include PGD_2_, PGE_2_, PGF_2α_, PGI_2_, and TXA_2_. They are released extracellularly immediately after their synthesis and they act by binding to a G-protein-coupled rhodopsin-type receptor on the surface of target cells. There are 8 types of prostanoid receptors that are conserved in mammals from mouse to human: the PGD receptor (DP), 4 subtypes of the PGE receptor (EP1, EP2, EP3, and EP4), the PGF receptor (FP), the PGI receptor (IP), and the TXA receptor (TP) [1].

Stevens-Johnson syndrome (SJS) and its severe variant, toxic epidermal necrolysis (TEN) are acute inflammatory vesiculobullous reactions of the skin and mucosa including the ocular surface [2]. In our earlier genome-wide association study in Japanese SJS/TEN patients with severe ocular surface complications we found associations with 6 single nucleotide polymorphisms (SNPs) in the prostaglandin E receptor 3 (EP3) gene (*PTGER3*) and we documented that compared with the controls, EP3 expression was markedly reduced in the conjunctival epithelium of SJS/TEN patients with severe ocular complications [3]. Others reported that the PGE_2_-EP3 signaling pathway negatively regulates allergic reactions in a murine allergic asthma model [4] and that it inhibits keratinocyte activation and exerts anti-inflammatory actions in mouse contact hypersensitivity [5]. We also showed that EP3 is dominantly expressed in the ocular surface-, especially the conjunctival epithelium, and that PGE_2_ acts as a ligand for EP3 in the conjunctival epithelium and down-regulates the progression of murine experimental allergic conjunctivitis [6]. In addition, we reported that an EP3 agonist suppressed the production of CCL5, CXCL10, CXCL11, and IL-6 in response to polyI:C stimulation of human conjunctival epithelial cells, suggesting that EP3 in the conjunctival epithelium may down-regulate ocular surface inflammation [7].

In the current study we investigated the expression of EP3 protein in the conjunctiva of patients with various ocular surface diseases such as SJS/TEN, chemical eye burns, Mooren’s ulcers, and ocular cicatricial pemphigoid (OCP).

## Materials and Methods

### Human conjunctival tissues

This study was approved by the Institutional Review Board of Kyoto Prefectural University of Medicine, Kyoto, Japan. All experiments were conducted in accordance with the principles set forth in the Helsinki Declaration.

Our immunohistochemistry controls were 3 nearly normal human conjunctival tissues acquired at surgery for conjunctivochalasis and one sample of normal conjunctival tissue acquired at limbal dermoid resection. Conjunctival tissues were also obtained from patients undergoing surgical reconstruction of the ocular surface due to SJS/TEN (n = 7), chemical eye burns (n = 3), OCP (n = 3), severe graft versus host disease (GVHD) (n = 1), pseudo-OCP (n = 1) and pterygium (PTG) (n = 1), from patients with Mooren's ulcers treated by resection of the inflammatory conjunctiva (n = 4), and from a patient with a giant papilla due to allergic vernal conjunctivitis. One conjunctival tissue sample was obtained from an SJS/TEN patient who did not require ocular surface reconstruction because ocular sequelae were minor (dry eye); this sample derived from additional unnecessary conjunctiva harvested just after cataract surgery.

### Immunohistochemistry

For EP3 staining we used rabbit polyclonal antibody to EP3 (Cayman Chemical Co., Ann Arbor, MI) [3], [6]. We previously checked and confirmed the EP3 specificity of this antibody using conjunctiva from EP3KO mice [6]. Further confirmation was by immunoblot analysis (Fig. S1). The secondary antibody (Biotin-SP-conjugated AffiniPure F(ab’)_2_ fragment donkey anti-rabbit IgG (H+L), 1∶500 dilution; Jackson Immuno Research, Baltimore, MD) was applied for 30 min, then VECTASTAIN ABC reagent (Vector Laboratories, Inc., Burlingame, CA) was added for increased sensitivity with peroxidase substrate solution (DAB substrate kit; Vector) as a chromogenic substrate.

### Evaluation of staining intensity using ImageJ software and down-regulation score

We converted the multi-color pictures into black and white pictures, and measured the gray value in the vertical line of the conjunctival epithelium. Then we recorded the average gray value on an intensity score from 5 to 16 (e.g. an average gray value of 100 was scored as 10). We also recorded the degree of down-regulation where "–"  =  intensity score 12–16, "+"  =  intensity score 8–11, and "++"  =  intensity score 5–7.

## Results

**Figure 1:**
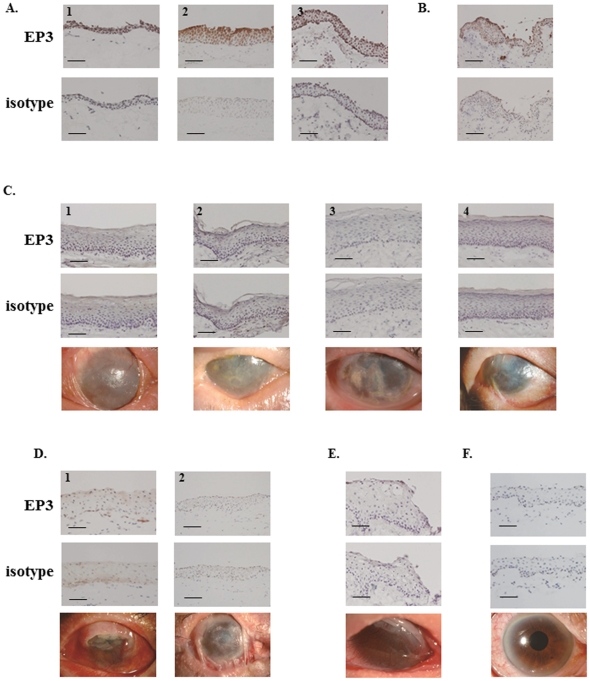
Immunohistological analysis of prostaglandin E receptor subtype EP3 in the conjunctival epithelium of the controls and SJS/TEN patients. A. Nearly normal conjunctival tissues from patients with conjunctivochalasis. B. Normal conjunctival tissue. C. Keratinized conjunctival tissues of SJS/TEN patients in the chronic stage. D. Non-keratinized conjunctival tissues of SJS/TEN patients in the sub-acute stage. E. Non-keratinized conjunctival tissues of SJS/TEN patients in the chronic stage. F. Visibly normal conjunctival tissue of an SJS/TEN patient with minor ocular sequelae (dry eye). C-F. The 3^rd^ lane shows the ocular surface of SJS/TEN patients. Each scale bar represents a length of 100 µm.

As reported elsewhere [3], EP3 protein was detected in the nearly normal conjunctival epithelium from patients with conjunctivochalasis (Fig. 1A) and in the normal conjunctival epithelium sample (Fig. 1B), but not in keratinized conjunctival epithelium from SJS/TEN patients in the chronic stage (Fig. 1C). When we examined non-keratinized conjunctival epithelium from SJS/TEN patients in the sub-acute- or chronic stage (Figs. 1D, 1E) we found that EP3 was markedly down-regulated. Interestingly, even in the conjunctival epithelium from the SJS/TEN patient manifesting only dry eye, EP3 was greatly down-regulated (Fig. 1F).

**Figure 2:**
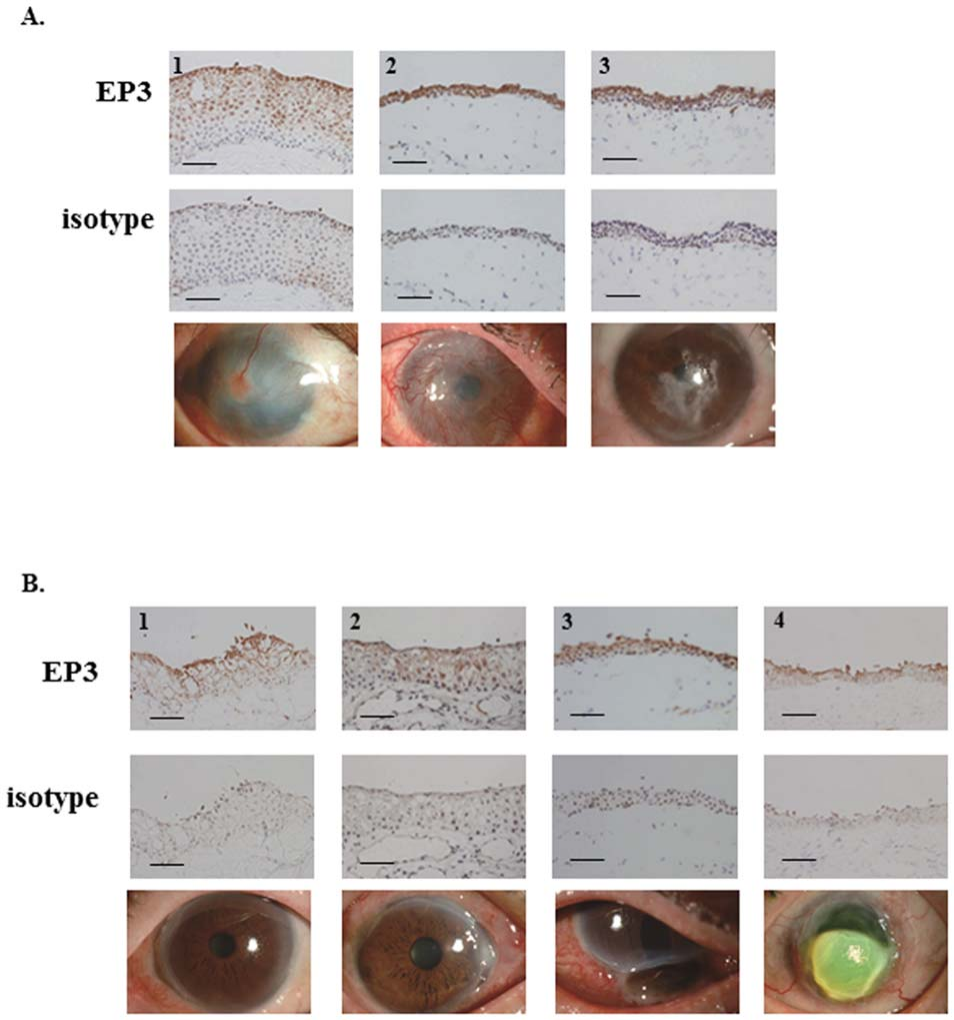
Immunohistological analysis of prostaglandin E receptor subtype EP3 in the conjunctival epithelium of patients with chemical eye burn and active Mooren’s ulcer. A. Conjunctival tissues of patients with chemical eye burn requiring ocular surface reconstruction. B. Inflammatory conjunctival tissues of patients with active Mooren’s ulcer requiring resection of the inflammatory conjunctiva. The 3^rd^ lane shows the ocular surface of patients. Each scale bar represents a length of 100 µm.

Comparison with conjunctival tissues from patients with chemical eye burn showed that although ocular surface findings were similar, EP3 protein was detected in the conjunctival epithelium of 3 patients with chemical eye burn as well as in control conjunctival epithelium from conjunctivochalasis patients (Fig. 2A). We also detected EP3 protein in conjunctival epithelium from 4 patients with Mooren’s ulcer, however, it appeared to be somewhat down-regulated (Fig. 2B).

**Figure 3:**
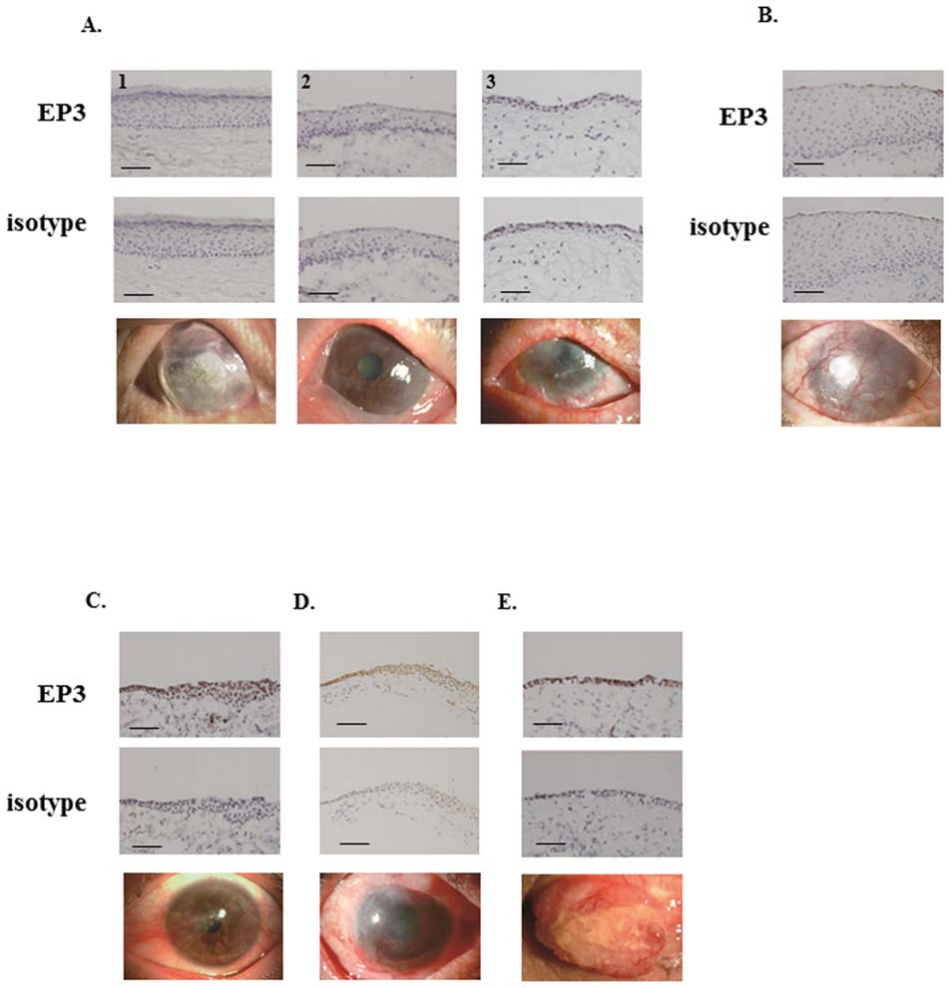
Immunohistological analysis of prostaglandin E receptor subtype EP3 in the conjunctival epithelium of patients with OCP (A), severe GVHD (B), pterygium (C), pseudo-OCP (D), and a giant papilla due to allergic vernal conjunctivitis (E). The 3^rd^ lane shows the ocular surface of patients. Each scale bar represents a length of 100 µm.

Next we examined conjunctival tissues from 3 patients with OCP; their ocular surface findings were very similar to those of SJS/TEN patients. No EP3 protein was detected in conjunctival epithelium from any of these patients (Fig. 3A), nor in conjunctival epithelium from a GVHD patient with severe conjunctival invasion to the cornea (Fig. 3B). When we assessed tissues from patients with pterygium (Fig. 3C), or pseudo-OCP (Fig. 3D), we detected EP3 protein in the conjunctival epithelium of pterygium patients as we did in the control conjunctival epithelium from a patient with conjunctivochalasis. EP3 protein was also present in conjunctival epithelium from patients with pseudo-OCP although it appeared to be slightly down-regulated. We also found EP3 protein in the conjunctival epithelium of a patient with giant papillae due to chronic allergic keratoconjunctivitis (Fig. 3E). In Table 1 we show the scores obtained by our evaluation of the staining intensity and degree of down-regulation for all samples.

**Table 1:** Staining-intensity score of conjunctival epithelium.

Picture Figure No		Intensity score	Down-regulation score	Disease
Figure 1	A1	14		Nearly normal conjunctival tissues from conjunctival chalasis
	A2	14		
	A3	14		
	B1	12		Normal conjunctival tissues
	C1	7	++	Keratinized conjunctival epithelium from SJS/TEN patients in the chronic stage
	C2	7	++	
	C3	6	++	
	C4	7	++	
	D1	5	++	Non-keratinized conjunctival epithelium from SJS/TEN patients in the sub-acute stage
	D2	5	++	
	E	5	++	Non-keratinized conjunctival epithelium from SJS/TEN patients in the chronic stage
	F	5	++	Conjunctival epithelium from an SJS/TEN patient manifesting only dry eye
Figure 2	A1	9	+	Chemical eye burn
	A2	16		
	A3	13		
	B1	12		Mooren’s ulcer
	B2	12		
	B3	14		
	B4	10	+	
Figure 3	A1	6	++	Ocular cicatricial pemphigoid (OCP)
	A2	6	++	
	A3	6	++	
	B	6	++	GVHD with severe conjunctival invasion to the cornea
	C	13		Pterygium
	D	10	+	Pseudo-OCP
	E	16		Chronic allergic keratoconjunctivitis

We document that EP3 was expressed in conjunctival epithelium of patients with chemical eye burns and Mooren’s ulcer and in normal human conjunctival epithelium. It was markedly down-regulated in the conjunctival epithelium of SJS/TEN- and OCP patients. Although we had only one patient each with severe GVHD, pterygium, pseudo-OCP, and chronic allergic keratoconjunctivitis, study of these samples suggested that EP3 is expressed in the conjunctival epithelium of patients with pterygium, pseudo-OCP, and chronic allergic keratoconjunctivitis, and that EP3 might be greatly down-regulated in the conjunctival epithelium of patients with severe GVHD.

Regarding in conjunctival epithelium, the expression of EP3 protein in the SJS/TEN and OCP patients was markedly decreased compared with normal conjunctiva. However, its expression in sub-conjunctival tissues may be up-regulated in some instances because vascular endothelia expressing the EP3 protein could be increased due to the presence of inflammatory infiltrating cells in sub-conjunctival tissues (Fig. S2).

## Discussion

We previously reported that in Japanese SJS/TEN patients there was a significant association between severe ocular surface complications and prostaglandin E receptor 3 gene (*PTGER3*) polymorphisms and that compared to the controls, EP3 expression was greatly reduced in their conjunctival epithelium [3]. Here we studied keratinized and non-keratinized conjunctival epithelia of SJS/TEN patients and the conjunctival epithelium of an SJS/TEN patient whose ocular sequelae were minor (dry eye). We found that EP3 was markedly down-regulated not only in keratinized- but also in non-keratinized conjunctival epithelia and even in the normal conjunctiva of a patient in the chronic stage of SJS whose only ocular sequela was dry eye. Our results suggest that the strong down-regulation of EP3 in conjunctival epithelium of SJS/TEN patients is associated with the pathogenesis and pathology of the disease because PTGER3 (EP3) polymorphisms are significantly associated with SJS/TEN.

Severe chemical eye burn results in conjunctival invasion into the cornea due to a deficiency in corneal epithelial stem cells; this leads to devastating ocular surface disorders similar to SJS/TEN. However, EP3 was not down-regulated in the conjunctival epithelium of patients with severe chemical eye burns, suggesting that the pathology of the ocular surface changes was not associated with EP3 expression.

In patients with Mooren’s ulcer the peripheral stroma is destroyed first circumferentially then centrally, resulting in the characteristic overhanging inner edge. This is an inflammatory disease of the ocular surface that may require resection of the inflammatory conjunctiva adjacent to the ulcer. We found that the conjunctival epithelium of the inflammatory conjunctival tissues adjacent to the ulcer clearly expressed EP3 protein, indicating that other factors besides inflammation are required for a marked down-regulation of EP3 expression.

OCP is a subset of mucous membrane pemphigoid. It is characterized by the abnormal production of circulating autoantibodies directed against various components of the basement membrane zone and the generation of proinflammatory and fibrogenic cytokines [8]. We found that, as in SJS/TEN patients, EP3 was markedly down-regulated in the conjunctival epithelium of OCP patients with conjunctival invasion to the cornea. As in OCP patients, we failed to detect EP3 protein in the conjunctival epithelium of a patient with severe GVHD with conjunctival invasion to the cornea. This suggests that in a common mechanism(s) may underlie the pathology of SJS/TEN and OCP, especially in ocular surface epithelium such as the conjunctival epithelium. EP3 expression has been reported in skin and PGE_2_ was produced abundantly during skin allergic inflammation [5], suggesting that there is no association between decreased EP3 expression and the increased production of cornified proteins in SJS/TEN and OCP.

We found that EP3 was clearly expressed in the conjunctival epithelium of our patients with pterygium, pseudo-OCP, and a giant papilla of allergic vernal conjunctivitis. Interestingly, the expression of EP3 in conjunctival epithelium from patients with OCP and pseudo-OCP was different: EP3 was clearly present in the patient with pseudo-OCP but not the patient with OCP. The patient with pseudo-OCP had received long-term treatment with eye drops for glaucoma; this resulted in a deficiency of corneal epithelial stem cells and led to conjunctival invasion into the cornea. This suggests that different mechanisms are involved in the expression of EP3. We also detected EP3 in the conjunctival epithelium of the patient with allergic vernal conjunctivitis. Elsewhere we documented that PGE_2_ acts as a ligand for EP3 in the conjunctival epithelium and down-regulates the progression of murine experimental allergic conjunctivitis [6]. Although EP3 may down-regulate allergic reactions in patients with allergic conjunctivitis, its loss may not be a causative factor.

In summary, EP3 is expressed not only in normal human conjunctival epithelium but also in the conjunctival epithelium of patients with chemical eye burns and Mooren’s ulcer. On the other hand, it is markedly down-regulated in the conjunctival epithelium of SJS/TEN- and OCP patients.

## Supporting Information

Figure S1 **The rabbit polyclonal antibody to EP3 we used is checked and confirmed the EP3 specificity of this antibody using immunoblot analysis.**
(TIF)

Figure S2 **EP3 expression in sub-conjunctival tissues in a SJS/TEN patient in the chronic stage.** In some instances of SJS/TEN patients, vascular endothelia expressing the EP3 protein are found.
(TIF)
