# Supplementary figures and images for: Prostaglandin E Receptor Subtype EP3 Expression in Human Conjunctival Epithelium and Its Changes in Various Ocular Surface Disorders
Source: PLoS One. 2011 Sep 22;6(9):e25209. doi: 10.1371/journal.pone.0025209 (PMC3178633; doi:10.1371/journal.pone.0025209)

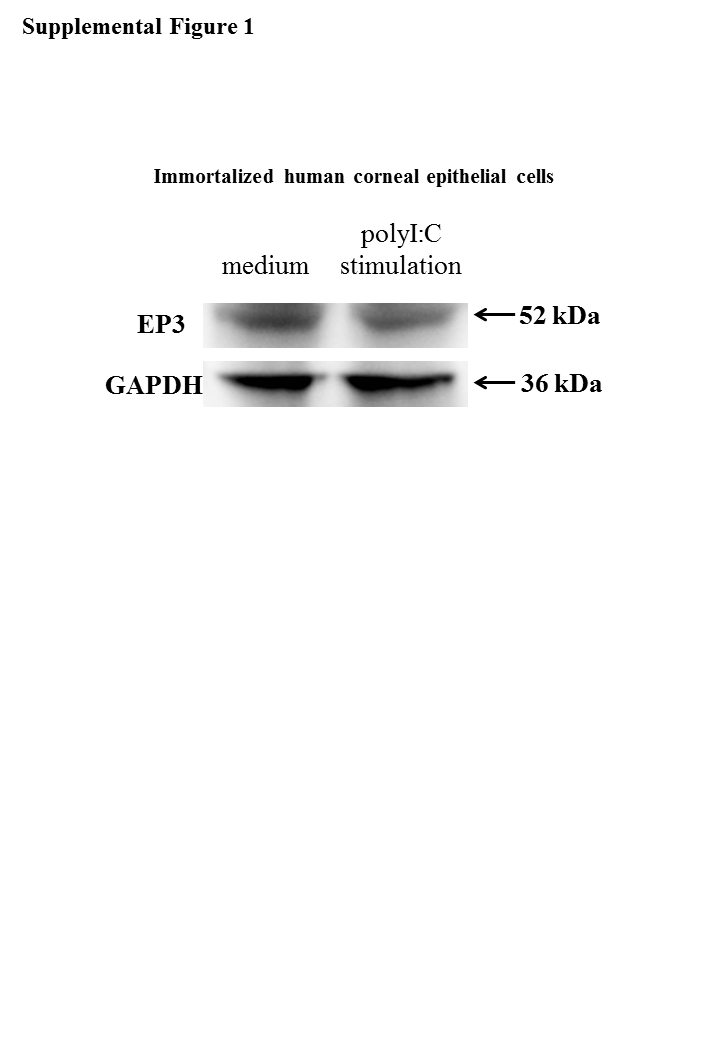

Supplement: Figure S1 — The rabbit polyclonal antibody to EP3 we used is checked and confirmed the EP3 specificity of this antibody using immunoblot analysis. (TIF) [file pone.0025209.s001.tif]

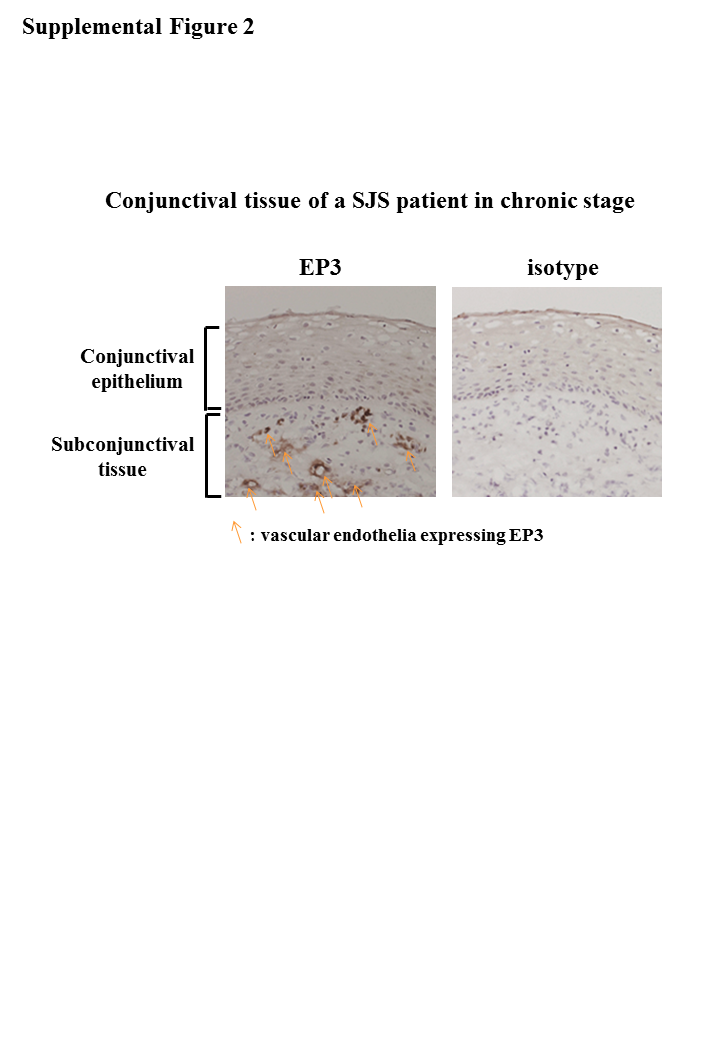

Supplement: Figure S2 — EP3 expression in sub-conjunctival tissues in a SJS/TEN patient in the chronic stage. In some instances of SJS/TEN patients, vascular endothelia expressing the EP3 protein are found. (TIF) [file pone.0025209.s002.tif]
